# Nanoscale programming of cellular and physiological phenotypes: inorganic meets organic programming

**DOI:** 10.1038/s41540-021-00176-8

**Published:** 2021-03-11

**Authors:** Nikolay V. Dokholyan

**Affiliations:** 1 Departments of Pharmacology, Penn State College of Medicine, Hershey, PA 17033-0850 USA; 2 Departments of Biochemistry & Molecular Biology, Penn State College of Medicine, Hershey, PA 17033-0850 USA; 3 Departments of Chemistry, and Biomedical Engineering, Penn State University, University Park, PA 16802 USA; 4 Departments of Biomedical Engineering, Penn State University, University Park, PA 16802 USA

**Keywords:** Protein design, Synthetic biology

## Abstract

The advent of protein design in recent years has brought us within reach of developing a “nanoscale programing language,” in which molecules serve as operands with their conformational states functioning as logic gates. Combining these operands into a set of operations will result in a functional program, which is executed using nanoscale computing agents (NCAs). These agents would respond to any given input and return the desired output signal. The ability to utilize natural evolutionary processes would allow code to “evolve” in the course of computation, thus enabling radically new algorithmic developments. NCAs will revolutionize the studies of biological systems, enable a deeper understanding of human biology and disease, and facilitate the development of in situ precision therapeutics. Since NCAs can be extended to novel reactions and processes not seen in biological systems, the growth of this field will spark the growth of biotechnological applications with wide-ranging impacts, including fields not typically considered relevant to biology. Unlike traditional approaches in synthetic biology that are based on the rewiring of signaling pathways in cells, NCAs are autonomous vehicles based on single-chain proteins. In this perspective, I will introduce and discuss this new field of biological computing, as well as challenges and the future of the NCA. Addressing these challenges will provide a significant leap in technology for programming living cells.

The history of programming dates back to nineth century when brothers Abū Jaʿfar, Muḥammad ibn Mūsā ibn Shākir, Abū al‐Qāsim, Aḥmad ibn Mūsā ibn Shākir, and Al-Ḥasan ibn Mūsā ibn Shākir, who have first described an automated flute in their Book of Ingenious Devices^1^. Since then, many inventions that automated instructions to perform a particular task were implemented on numerous platforms, including biological materials. Perhaps the most notable example was the experiment by Luigi Galvani in XVIII century, who controlled the contraction of detached frog legs using an electric current^2^. Since then the electric control of live matter moved to tissue level with such notable applications as cardiac pacemakers^3^, brain^4^, and vagus nerve^5^ stimulators. Most recently, the emergence of the computer-brain interface is enabling “read and write” brain signals in a desirable fashion, thereby enabling control over arterial blood pressure^6^, restoration of the motor functions after stroke^7^, and conscious brian-to brain communication in humans^8^. The emergence of the field of synthetic biology^9–11^ moved the control to a single cell level. The programming, as we know it today, has undergone radical evolution in nineteenth century with the invention of the silicon-based computers. Bioprogramming, like silicon-based coding, is a set of instructions aimed to achieve a particular task, but unlike silicon-based programming, these instructions are operated on biological molecules, such as DNA, RNA and proteins, and aimed at manipulation of specific phenotypic output in living cells.

We are on the threshold of creating nanoscale cellular computers using biological molecules for bioprogramming cellular phenotypes. The revolution in the field of protein design^12–14^ has allowed us to establish rational control of proteins in living cells. With this progress, we are within reach of developing a “nanoscale programming language”, in which molecules serve as operands, and their conformational states function as logic gates. Combining these operands through protein engineering into larger molecules and molecular complexes will allow us to write and execute “code” using NCAs. As with other computer languages, these agents would respond to input and return output signals. While the speed of the “computation” would be significantly slower than that of inorganic silicon-based computers, one cell could contain more computational agents than the number of CPUs in a supercomputer. Furthermore, the ability to utilize natural evolutionary processes would allow code to “evolve” in the course of computation, thus enabling radically new algorithmic developments.

While this vision may sound like science fiction, a number of elements of this technology already exist, and several laboratories have executed some of these programs, fueling the emergence of the field of synthetic biology. These elements include approaches to sense and control proteins in living cells. Streamlined nanoscale biological computation, *bioprogramming*, will allow direct interrogation of biological systems, enable a deeper understanding of human biology and disease, and introduce possibilities for precision therapeutics. Furthermore, since bioprogramming can be extended to novel reactions and processes not typically seen in biological systems, growth of this field will spark the development of biotechnological applications with impact outside of biological fields. Unlike traditional approaches in synthetic biology that are based on rewiring/hijacking signaling pathways in cells^15–21^, NCAs are autonomous vehicles based on single-chain proteins or, plausibly in the future, RNA molecules. Although NCAs are susceptible to expression variability, they present a single expression variable compared to that of multicomponent circuit rewiring, done in classical synthetic biology approaches. NCAs offer a complementary mean for controlling cellular phenotypes. Importantly, the size of the “program” (i.e., DNA code) based on NCA is drastically smaller than that of programs utilizing synthetic biology approaches. Such code “compression” is possible due to direct design of a protein function rather than indirect control of it through protein expression, as it is done in synthetic biology approaches.

The main component of the NCA is the response unit (RU) – a protein whose output is a biological signal (Fig. 1). RUs are akin to computer motherboards with attached outputs. The input to the RU can be provided by a number of functional modulators (FMs), such as light- or drug-sensitive functional modulators (LFMs and DFMs), as well as other specialized units, such as pH-sensitive, temperature-sensitive, and/or RNA sensitive units. The input domains can be combined to produce a complex response by RUs. In Fig. 1, two DFMs are combined in one so that the input from them generates a signal of higher complexity than that produced by one DFM: this combined DFM can respond to ligands A, B, and A and B (e.g., A and B could be brought separately or as one when connected via a (potentially cleavable) linker). In addition, LFM is “wired” through steric or allosteric networks to influence both output functions *F*(*x*) and *G*(*x*) of the RU (here, *x* is the input vector). Examples of the output can be catalysis, (de)activation, homo- or hetero-dimerization, oligomerization, localization, translocation and many other desired functions performed by proteins in cells. The output is generated either through conformational changes in the RU unit or changes in the dynamics of the RU’s active site. For example, in Fig. 1 function *F*(*x*) depends on the surface of the RU that interfaces other binding partners, while function *G*(*x*) regulates the active site dynamically without changing the conformation of the RU. While the output can be conceived as a binary response, this response can be fine-tuned to adapt to a desired dynamic range.

**Fig. 1:**
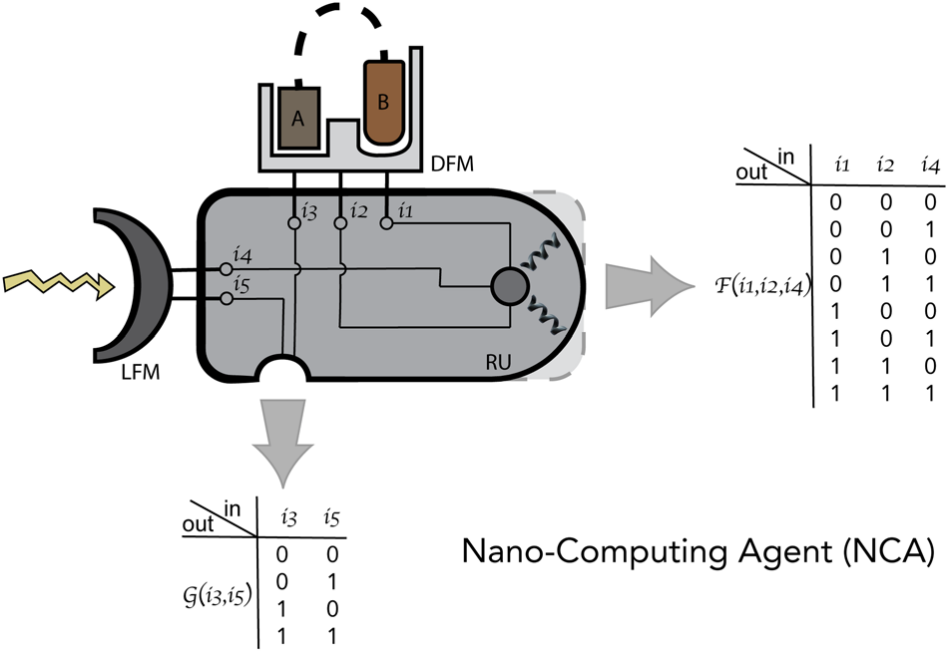
A conceptual diagram of the NCAs. The response unit (RU) is controlled by light- or drug-sensitive regulatory units (LFM, DFM). The control is established either via allosteric networks within proteins or through direct steric interactions. The inputs $\left\{ {{\mathrm{i}}_1, \ldots ,{\mathrm{i}}_5} \right\}$ and the outputs F and G are represented as binary functions for simplicity.

There are two principal requirements for using NCAs in cells. First, they must be “stealthy”^22^, meaning that the NCAs do not affect the cellular phenotype without activation of LFMs and DFMs, and that RU behaves as if it did not have regulatory units attached. To address this requirement, we can utilize protein allostery to regulate protein function^22,23^. In this way, we will be able to avoid functionally important protein surfaces. Second, for simplicity and consistency of operation, the NCAs must be genetically encoded and introduced to cells either via transient transfection or generating a stable cell line.

The conceptual architecture of an NCA is as follows: the main unit RU is a protein or a protein domain, capable of multiple responses, such as alterations of (i) surfaces used to bind other proteins, (ii) active site structure, (iii) dynamics of the active site, (iv) post-translational modifications, and (v) other conformational changes that result in altered function of this protein. This RU is controlled by functional modulators responding to light, drug, pH, temperature, RNA, or any other user-defined input. The wiring of these functional modulators is performed through allosteric networks^24,25^ or direct steric gating^26^. In the latter, FM can be used to sterically interfere with the activity of the RUs. In the former, it is possible to utilize dynamic allostery^27^ (Fig. 2); whereby, upon ligand binding, the active site exhibits altered dynamics thus affecting the RU’s function. In the process of ligand binding, RUs maintain structural equivalence of active versus inactive states of the unmodified RU, thereby limiting interference of our FMs with the RU’s endogenous interaction partners, and maintaining stealthy control over the RUs’ active sites^22,24^.

**Fig. 2:**
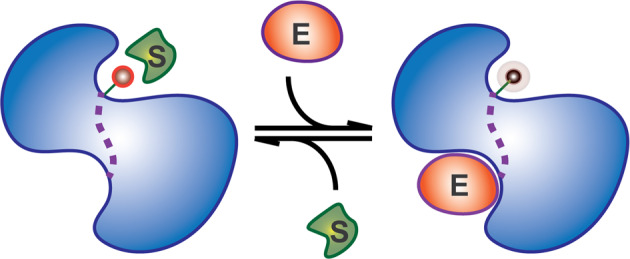
Schematic diagram of dynamic allostery. An effector, E, binding to an enzyme results in increased dynamics of the active site residues, reducing the likelihood of interaction with the substrate, S, and thus reducing the enzymatic activity of the protein.

Some of the established modes of controlling RUs are photo/chemo-allosteric activation and inhibition (Fig. 3). Other modes, such as steric gating^26^ and the controlled split protein reassembly method (SPELL)^28^ (Fig. 3D), offer additional methods of bioprogramming RUs. The proof of concept of simultaneous, multiplexed control of proteins in cells by modulating several RUs at the same time, was recently demonstrated by Dagliyan et al.^29^

**Fig. 3:**
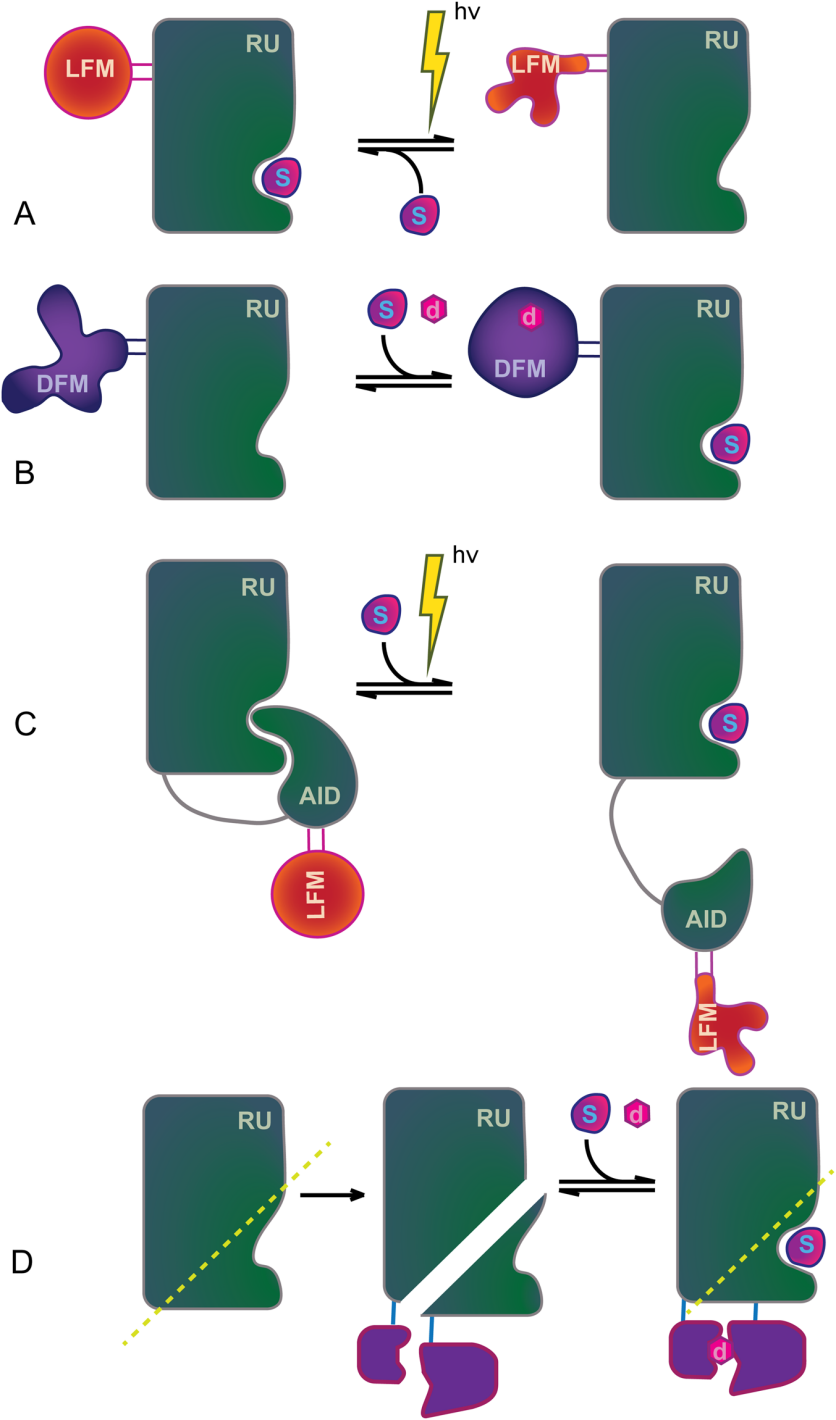
Some of the established modes of protein control^26,27^. **A** Photo-allosteric inhibition (e.g., using LOV2): upon irradiation with light, disorder induced in LOV2 (LFM) allosterically induces increased fluctuations in the active site, thereby inhibiting interaction with the substrate, S. **B** Chemo-allosteric inhibition: an engineered DFM (e.g., uniRapR) undergoes disorder-order transition upon addition of a small molecule (rapamycin); thereby, allosterically promoting interaction with the substrate. **C** Photo-allosteric activation: similar to **A** but LFM is inserted into autoinhibitory domains (AID). Upon irradiation by light, AID dissociates thereby activating the RU. Similarly, chemo-inhibition can be accomplished by targeting autoinhibitory domains (AID) by the drug-controlled domain uniRapR. **D** Controlling protein function via split reassembly^28^. An RU split into N and C parts that are functionalized with two conditionally dimerizing proteins (e.g., iFKBP and FRB). iFKBP is a designed^44^ FKBP variant that is predominantly disordered^47^, thereby disallowing spontaneous reassembly of N and C parts. Upon addition of rapamycin, iFKBP and FRB dimerize, stabilize iFKBP and bring N and C parts to form functional RU.

The other critical component of NCAs is a set of FMs or sensors (Fig. 4). Several groups have already developed and utilized light^30–39^ and drug-based sensors^40–46^. Two or more FMs can be combined to regulate complex RUs: multidomain proteins provide a rich platform for functionalization with multiple MFs. RUs themselves can be also combined to create an even richer platform. Perception of external conditions, such as temperature and pH, are critical to all species. Nature has adapted many hierarchical mechanisms for sensing these conditions: from molecules that undergo conformational change, or shape change^47^, to signaling within and between cells and organs. The sensing of conditions, as well as of molecules, is an important and critical step for developing a versatile palette of NCAs. Following our strategy of allosteric modulation of the RUs, we require that for designed FMs: (i) the C- and N- termini of FMs must be within 7–12 Å distance^29^, and (ii) the pH, temperature, or binding to other molecules must not destabilize the RUs. It is possible to utilize natural proteins that respond to pH, temperature and binding to molecules, as insertable scaffolds.

**Fig. 4:**
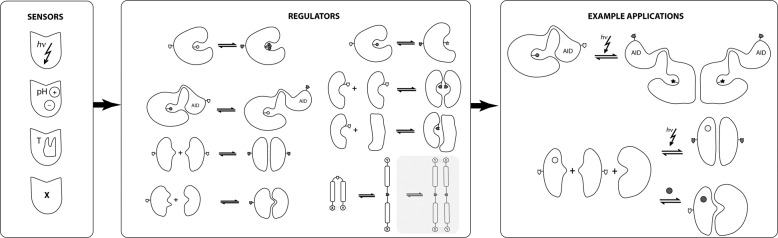
Bioprogramming in action: A suite of disparate sensors (FMs) can be functionalized to RUs which can respond upon FM stimulations by functional activation/inactivation, hetero/homo-dimerization, conformational switching and many other potential responses. By combining RUs and FMs, we can assemble combinatorial number of possible applications with desired functions.

Construction of NCAs will offer a novel direction in our ability to interrogate cellular and organismal life, and build novel pharmaceutical strategies. Among many future applications, we could pursue therapeutic interventions using NCAs by exploiting innate evolutionary pressure. For example, NCA may be designed to target kinases whose hyperactivity contributes to cancer. Failure of chemotherapy treatments often happens due to evolutionary adaptation of these kinases to drugs (e.g., via mutations in the drug-binding site). Adaptive changes of the designed NCA may be able to counter changes in kinases to keep them inactive. This example signifies radical new possibilities for establishing perpetual and autonomous regulation of proteins in living cells using NCAs.

## Challenges

To fully enable nanoscale biological computation – *bioprogramming* – we need to: (i) expand the repertoire of inputs; (ii) include other biological molecules (e.g., RNA, lipids, DNA, macrocycles, metabolites) to aid or perform computation; and (iii) expand the portfolio of approaches for “writing” algorithms at the nanoscale level. Mapping allosteric communications within proteins has been a focus of many laboratories. A number of methods have been to accurately map allosteric pathways^24,48,49^ and even had success in disrupting allosteric connections within proteins^50,51^. Designing of specific allosteric communications within proteins is a critical next challenge. While within reach, the technology to “rewire” allosteric networks in proteins has yet to be developed. Addressing these challenges will provide a significant leap in technology for programming living cells, and create a new direction in bioprogramming.
